# Meaningful Clinical Impact of ABvac40 in Early‐Stage Alzheimer’s Disease: Results from the AB1601 Phase 2 Study

**DOI:** 10.1002/alz70859_102589

**Published:** 2025-12-25

**Authors:** María Pascual‐Lucas, Ana María Lacosta, María Montañés, Jesús Canudas, Jorge Loscos, Gerard Piñol‐Ripoll, Jose Terencio, Mercè Boada

**Affiliations:** ^1^ Araclon Biotech, Zaragoza, Zaragoza Spain; ^2^ Araclon Biotech‐Grifols, Zaragoza Spain; ^3^ Unitat Trastorns Cognitius, Clinical Neuroscience Research, Santa Maria University Hospital, IRBLleida, Lleida Spain; ^4^ Hospital Clínic de Barcelona, Barcelona Spain; ^5^ Grifols S.A., Barcelona, Barcelona Spain; ^6^ Ace Alzheimer Center Barcelona‐Universitat Internacional de Catalunya, Barcelona Spain; ^7^ Networking Research Center on Neurodegenerative Diseases (CIBERNED), Instituto de Salud Carlos III, Madrid Spain

## Abstract

**Background:**

ABvac40 is an investigational vaccine targeting Aβ40 for the treatment of Alzheimer’s disease (AD). Aβ40 plays a significant role in several pathological processes of AD, especially concerning amyloid deposition in blood vessels. Phase 2 trial results (NCT03461276) demonstrated a favorable safety profile, robust immunogenicity, and promising trends in cognitive outcomes and brain atrophy. To further explore the therapeutic potential of ABvac40, we aimed to assess clinical relevance at individual patient level by evaluating meaningful within‐patient changes (MWPC). This approach examines the proportion of patients experiencing significant deterioration across treatment groups, providing a method to assess the meaningfulness of treatment effects in progressive conditions like AD.

**Method:**

AB1601 was a randomized, double‐blind, placebo‐controlled phase 2 study in patients with mild cognitive impairment or very mild AD. MWPC was defined as a decline of ≥3 points in MMSE at two consecutive visits over 24 months. Cox proportional hazard models assessed the risk of MWPC, adjusting for MMSE baseline score, baseline age, APOE Ɛ4 carrier status, clinical stage and baseline use of AD medication. Patients treated with ABvac40 were stratified by CSF antibody levels into two groups: high levels (Q4) and low levels (Q1–Q3). Analyses were conducted in the PPc analysis set.

**Result:**

A total of 97 patients were included in the PPc analysis set. Of these, 69 (71.1%) were amyloid‐PET positive. In the overall population, ABvac40 significantly reduced the risk of MWPC over 24 months compared to placebo (HR=0.47; 95% CI 0.27–0.85; P=0.012). In the amyloid‐PET positive subgroup, ABvac40 showed a more pronounced protective effect (HR=0.38; 95% CI 0.20‐0.75; P=0.005). Within this subgroup, patients with lower antibody levels had a 63% lower risk of MWPC (HR=0.37; 95% CI 0.16–0.83; P=0.016), while those with higher antibody levels showed an 81% reduction (HR=0.19; 95% CI 0.04–0.84; P=0.029).

**Conclusion:**

ABvac40 treatment significantly reduced clinically meaningful cognitive decline, particularly in amyloid‐PET positive patients, with the strongest effects in those with the highest antibody levels. These findings highlight ABvac40’s potential to influence disease progression in early‐stage AD and warrant further studies to explore its impact on brain vessels as potential driver of the observed cognitive benefits.

